# Emotional Analysis of Tweets About Clinically Extremely Vulnerable COVID-19 Groups

**DOI:** 10.7759/cureus.29323

**Published:** 2022-09-19

**Authors:** Toluwalase Awoyemi, Kayode E Ogunniyi, Adedolapo V Adejumo, Ujunwa Ebili, Abiola Olusanya, Eloho H Olojakpoke, Olufunto Shonibare

**Affiliations:** 1 Nuffield Department of Women’s and Reproductive Health, University of Oxford, Oxford, GBR; 2 Internal Medicine, University Hospital of North Durham, Durham, GBR; 3 Intensive Care Unit, Chelsea and Westminster Hospital NHS Foundation Trust, London, GBR; 4 Family Medicine, Emel Hospital, Lagos, NGA; 5 Department of Haematology, University Hospitals Coventry and Warwickshire, Coventry, GBR; 6 College of Medicine, University of Ibadan, Ibadan, NGA; 7 Internal Medicine, Gbagada General Hospital, Lagos, NGA

**Keywords:** sentiment analysis, twitter, highriskcovid, cevs, face masks, healthcare policy, covid-19

## Abstract

Background

Clinically extremely vulnerable (CEV) individuals have a significantly higher risk of morbidity and mortality from coronavirus disease 2019 (COVID-19). This high risk is due to predispositions such as chronic obstructive pulmonary disease (COPD), diabetes mellitus, hypertension, smoking, or extreme age (≥75). The initial COVID-19 preventive measures (use of face masks, social distancing, social bubbles) and vaccine allocation prioritized this group of vulnerable individuals to ensure their continued protection. However, as countries start relaxing the lockdown measures to help prevent socio-economic collapse, the impact of this relaxation on CEVs is once again brought to light. In this study, we set out to understand the impact of policy changes on the lives of CEVs by analyzing Twitter data with the hashtag #highriskcovid used by many high-risk individuals to tweet about and express their opinions and feelings.

Methodology

Tweets were extracted from the Twitter API between March 01, 2022, and April 21, 2022, using the Twarc2 tool. Extracted tweets were in English and included the hashtag #highriskcovid. We evaluated the most frequently used words and hashtags by calculating term frequency-inverse document frequency, and the location of tweets using the tidygeocoder package (method = osm). We also evaluated the sentiments and emotions depicted by these tweets using the National Research Council sentiment lexicon of the Syuzhet package. Finally, we used the latent Dirichlet allocation algorithm to determine relevant high-risk COVID-19 themes.

Results

The vast majority of the tweets originated from the United States (64%), Canada (22%), and the United Kingdom (4%). The most common hashtags were #highriskcovid (25.5%), #covid (6.82%), #immunocompromised (4.93%), #covidisnotover (4.0%), and #Maskup (1.40%), and the most frequently used words were immunocompromised (1.64%), people (1.4%), disabled (0.97%), maskup (0.85%), and eugenics (0.85%). The tweets were more negative (19.27%) than positive, and the most expressed negative emotions were fear (13.62%) and sadness (12.47%). At the same time, trust was the most expressed positive emotion and was used in relation to belief in masks, policies, and health workers to help. Finally, we detected frequently co-tweeted words such asmass and disaster, deadly and disabling, high and risk, public and health, immunocompromised and people, mass and disaster, and deadly and disabling.

Conclusions

The study provides evidence regarding the concerns and fears of high-risk COVID-19 groups as expressed via social media. It is imperative that further policies be implemented to specifically protect the health and mental wellness of high-risk individuals (for example, incorporating sentiment analyses of high-risk COVID-19 individuals such as this paper to inform the evaluation of already implemented preventive measures and policies). In addition, considerable work needs to be done to educate the public on high-risk individuals.

## Introduction

Coronavirus disease 2019 (COVID-19) is a viral respiratory and multi-systemic illness that emerged in China and has ravaged the world as a pandemic since late 2019 with rapid spread and mounting mortality figures. This rapid spread and the significant morbidity and mortality forced governments worldwide to impose stringent public health measures to curb the spread of the disease [1]. In January 2022, the United Kingdom removed virtually all COVID-19 restrictions (including vaccine passports) and reduced the number of places where the use of face masks is mandatory [2,3]. This was replicated by the United States, which reviewed its COVID-19 mandate to enable up to 90% of the population to move around without masks [4]. Similarly, Ghana abolished compulsory mask use on March 28, 2022 [5]. This sparked outrage worldwide [6,7], especially among clinically extremely vulnerable (CEV) individuals and their caretakers. CEV refers to individuals who, due to specific health conditions, predispositions, or malignancy, are at a heightened risk of more severe manifestations of COVID-19 and death [8-16]. Some examples of CEVs are those in older age groups (≥75 compared to <65 years), males, smokers, chronic obstructive pulmonary disease (COPD) patients, diabetic patients, hypertensive patients, those with hematological malignancies, and solid organ transplant recipients. The complete list can be found in Appendices (Table 1).

Most CEV individuals are anxious about their deteriorating mental health, safety, physical health, isolation, and the attitude of the general immunocompetent population toward the pandemic [17]. Therefore, various governmental agencies prioritized the care of these high-risk groups by granting them access to vaccinations, medical care, and social amenities [18]. There were rapid COVID-19 preventive measures, masking mandates to reduce transmission, restriction of international trade and travel, and vaccination to protect households with high-risk individuals [19]. Eventually, these restrictions and guidelines resulted in a spectrum of reactions from different strata of society as the socio-economic implications and uncertainty of an end date started to rise [20,21]. Governments and their agencies had difficult choices to make: either continue lockdown measures or ease off. Likewise, citizens had less than palatable choices: either protect the vulnerable and accept the restrictions as the new norm or mount pressure on the government to eliminate COVID-19 limits irrespective of the consequences to high-risk groups.

As the socio-economic implications of the imposed restrictions began to manifest, governments lifted the COVID-19 restrictions in phases. Not surprisingly, this came with attendant opinions and sentiments, with reactions being very pliable and changing in response to the twists and turns of the pandemic [22-24]. One famous outlet for opinions and grievances was Twitter. Millions of people on this social media platform interact quasi-anonymously to share views and arguments, communicate their feelings minute-to-minute, and receive feedback. Unfortunately, this outlet representing enormous data that can help guide and direct governmental and population-based interventions has been underutilized. Data from Twitter (and other social media platforms) can be easily accessed and probed by sentiment analysis, a process that has been used in business and human resource settings with real-life ramifications but is relatively new in the healthcare space [25-27]. In this paper, we analyzed the sentiments and emotions around the Twitter hashtag #highriskcovid, which is about individuals at high risk of severe COVID-19. The aim is to provide a much-needed and timely patient perspective and help identify the most relevant problems faced by high-risk individuals to help direct and target population-based public health strategies.

## Materials and methods

We extracted relevant tweets for this analysis using the Twitter streaming application programming interface and pre-processed the data by cleaning, filtering, and analyzing adequately to test our hypothesis.

Data collection

We extracted all English-language tweets related to #highriskcovid with the Twarc2 tool on power shell from March 1, 2022, to April 21, 2022. We chose this duration arbitrarily based on the most recent use of the hashtag till the time of analysis. The hashtag had previously been used three times at different time points in the pandemic, but the fourth use coincided with the relaxing preventive measures. Thus, we believed analyzing tweets during this period would be sufficient to capture the reaction of people. We processed and converted the output file (JSON file) by flattening and converting it into a comma-delimited file (comma-separated values, CSV). All subsequent analysis on the processed CSV file was done in R.

Data analysis and visualization

Data Loading

The CSV file was deduplicated from 10,625 total tweets initially to 1,348 unique tweets.

Data Pre-processing

We created a custom R code to pre-process filtered tweets to remove irrelevant components of tweets (URLs, retweets, numbers, emojis, special characters, graphs, symbols such as @, /, //, digits, numbers, white spaces, websites, and any identifiable information, hyperlinks, and hash symbols). We extracted the text, location, retweet counts, likes, and quoted tweets and converted them to a corpus (text mining structure). We transformed all components of the corpus to lowercase to reduce ambiguity. We excluded known and custom (such as corona or viruses that may relate to other topics) stopwords with the TM package. Finally, we stemmed the corpus to reduce the words to their root.

Descriptive Analysis

We extracted relevant metrics (likes, quoted tweets, retweets, and replies) and analyzed the data with the psych package.

Frequency of Words and Hashtags

We subsequently determined the word importance in our tweets by calculating term frequency-inverse document frequency (TF-IDF). This process generated a document-term matrix. We analyzed and visualized the frequencies of the most common words with a bar plot.

For the hashtag analysis, we extracted the hashtags associated with the tweets and pre-processed them as previously described, which included converting them to a text corpus. We analyzed the most frequent terms with the qdap package.

Location Analysis

We extracted the author location of our tweets and reverse geocoded them to obtain the latitude and longitude of the locations with the tidygeocoder package (method = osm). We did this to ensure uniformity of location as some tweet authors had used different location depictions (city, state, or country). The coordinates were converted to countries with the sp and rworldmap packages using the coords2country function.

Sentiment and Emotion Quotient Analysis

We performed a sentiment and emotion quotient analysis on our tweets with the get_nrc_sentiment function of the Syuzhet package using the National Research Council (NRC) sentiment lexicon. The NRC sentiment lexicon consists of eight different emotions, namely, trust (positive), surprise (positive or negative), sadness (negative), joy (positive), fear (negative), disgust (negative), anticipation (positive or negative), and anger (negative). The NRC sentiment lexicon is widely utilized in sentiment analysis and represents the only lexicon that allows simultaneous analysis of emotions in addition to sentiments.

Latent Dirichlet allocation model building

**Figure 1:**
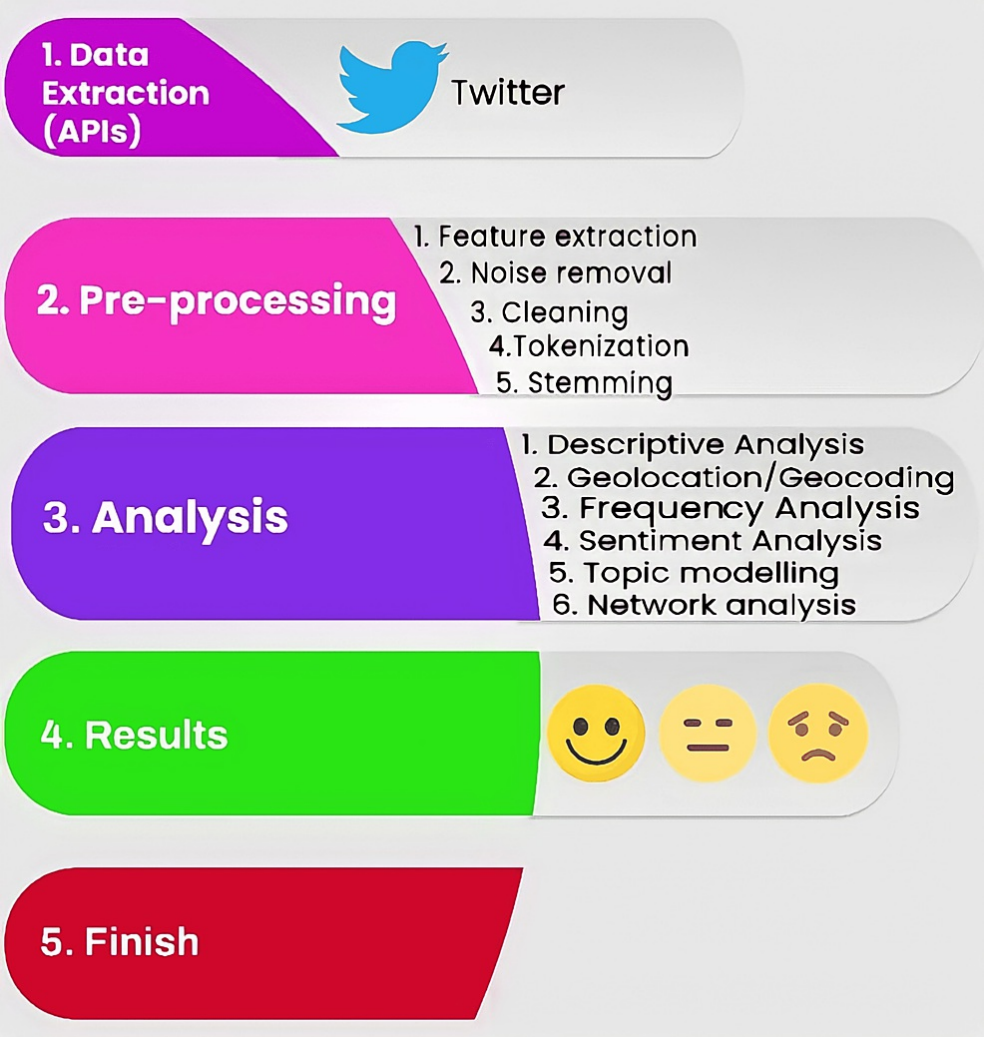
The analytic workflow used in this paper.

Finally, we used a latent Dirichlet allocation (LDA) algorithm (an unsupervised machine learning analysis) to discern clusters of keywords and identify common topics and themes. Before fitting the LDA model, we finetuned the LDA model by calculating perplexity scores with the ldatuning package. We divided the dataset into five subsets (one training subset and four test subsets) and performed validation and cross-validation on a series of k (topic number) from 2 to 50 (burn in = 1,000, iteration = 1,000, keep = 50). The corresponding perplexity score was graphed and inspected (Appendices, Figure 13) and used to inform the choice of k for the FindTopicsNumber in ldatuning. We finetuned the LDA model by running the Gibbs algorithm and serially analyzing k from 2 to 40 (Appendices, Figure 14) with Griffiths2004 and Arun2010 metrics (seed = 77). We then selected 15 as the optimal topic number for our LDA model. We fitted the data to the LDA model (seed = 1,234, burn in = 1,000, iteration = 1,000, keep = 50, alpha = 0.1). We depicted the top four topics identified and their associated words (top 10) as bar graphs. Finally, we visualized the bigram count network (network of words that are frequently tweeted together) using the network3D package as a node map and set the weight threshold set at 50. Figure 1 contains the summary of the analytic steps performed.

## Results

Descriptive analysis of high-risk COVID-19 tweets

On the first attempt, we sourced 10,652 tweets. After removing duplicates, we had 1,348 tweets left. Of the tweets analyzed, the minimum number of likes per tweet was zero, while the maximum number of likes on a tweet was 14,654. The median likes per tweet were two. Similarly, the minimum number of quotes per tweet was zero, the maximum per tweet was 322, and the median number of quotes was zero. Finally, the minimum number of replies per tweet was zero, the maximum number of replies per tweet was 450, and the median replies per tweet were zero.

**Figure 2:**
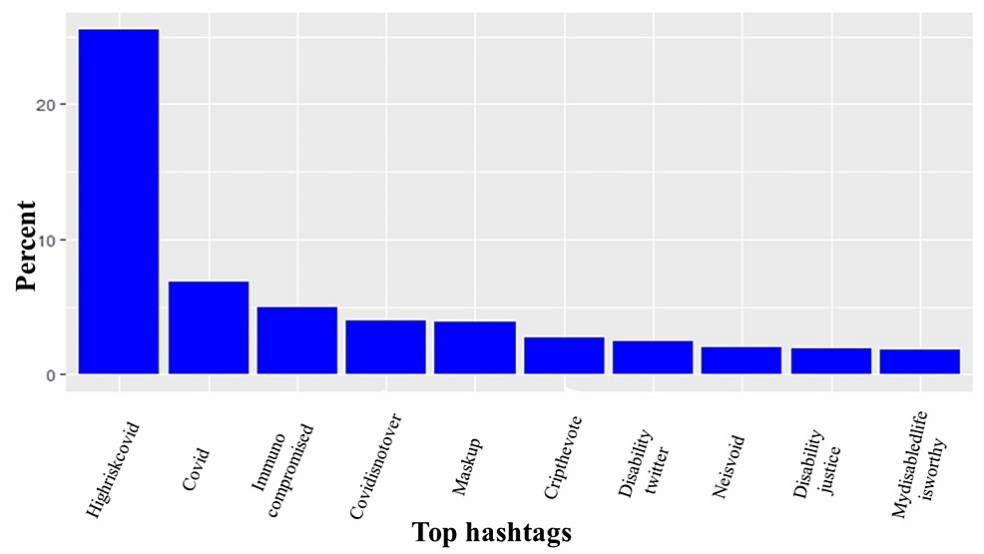
Top 10 hashtags used in association with #highriskcovid tweets. The Y axis is the percentage of each hashtag relative to all analyzed tweets, while the X axis contains the top 10 hashtags associated with #highriskcovid tweets.

**Figure 3:**
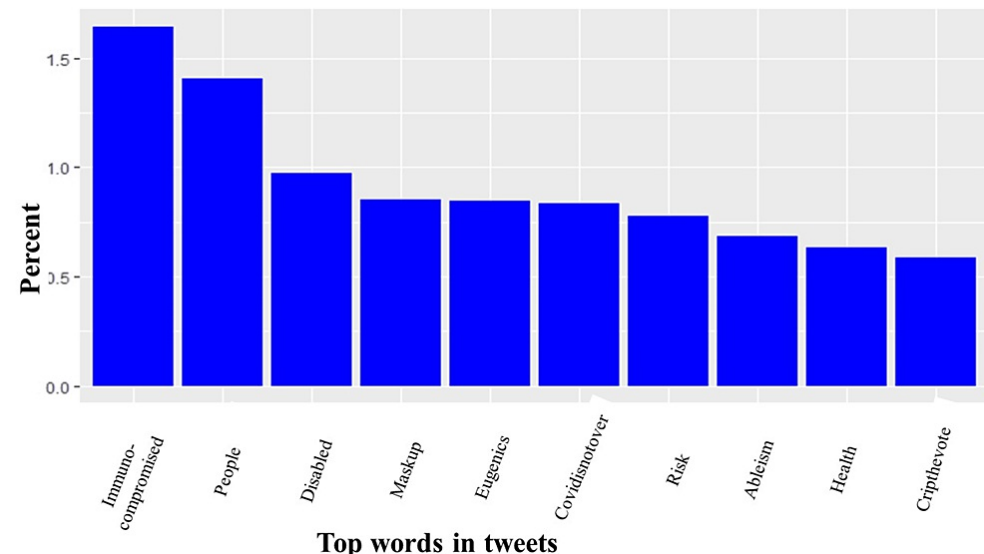
Top 10 words used in #highriskcovid tweets. The Y axis is the percentage of each word relative to all analyzed tweets, while the X axis contains the top 10 words used in #highriskcovid tweets.

**Figure 4:**
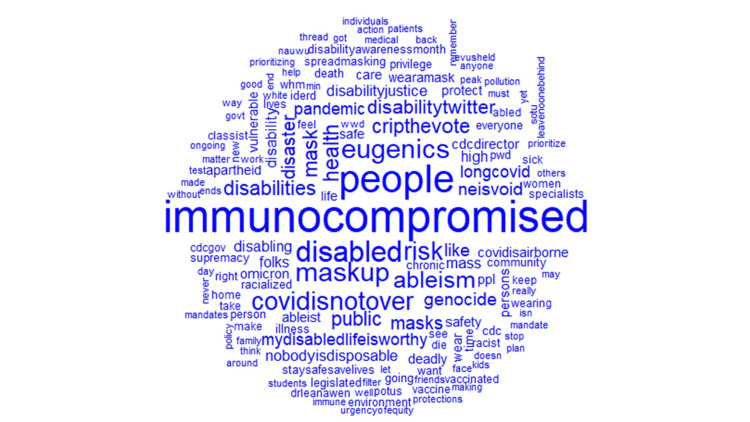
Word cloud of #highriskcovid words with a frequency of occurrence greater than 25.

**Figure 5:**
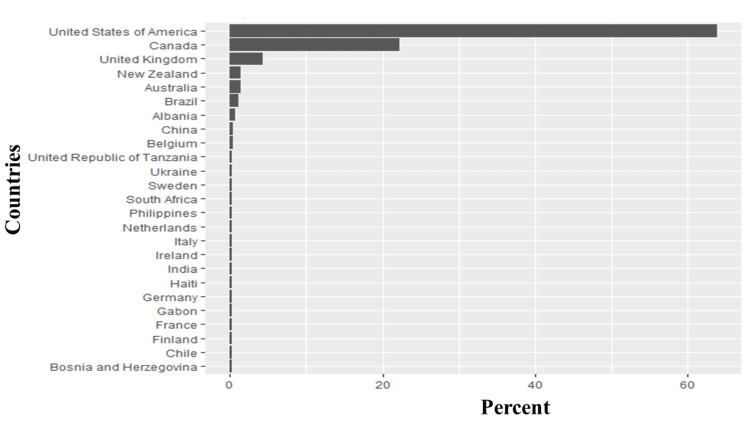
Countries of origin of tweets analyzed in this study. The Y axis contains the top 25 countries with the most #highriskcovid tweets, while the X axis is the percentage of each country relative to the whole tweet.

The most common hashtags were #highriskcovid (25.5%), #covid (6.82%), immunocompromised (4.93%), #covidisnotover (4.0%), and #Maskup (1.40%), with #highcovid being the most frequently occurring hashtag associated with tweets (Figure 2). Further analysis showed that words such as immunocompromised (1.64%), people (1.4%), disabled (0.97%), maskup (0.85%), and eugenics (0.85%) frequently occurred in high-risk COVID-19 tweets. Figure 3 shows the top 10 frequently tweeted words. Figure 4 is a word cloud of the top 100 words and includes words such as ableism, genocide, disabilitytwitter, longcovid, nobodyisdisposable, and supremacy. Of the tweets where location data were available, 25 countries were represented, and the vast majority of tweets originated from the United States (64%), Canada (22%), and the United Kingdom (4%) (Figure 5).

Sentiment and emotional quotient analysis of high-risk COVID-19 tweets

**Figure 6:**
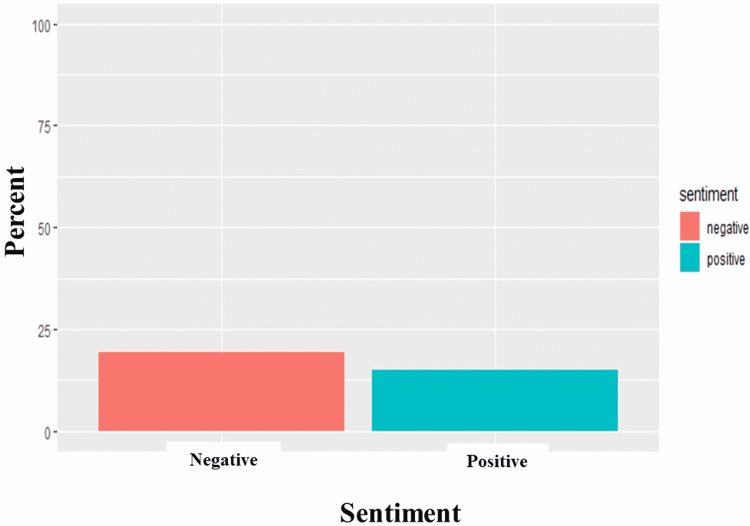
Sentiment analysis of #highriskcovid tweets. The Y axis is the percentage of sentiments of all analyzed tweets, while the X axis refers to the sentiments (negative or positive) in #highriskcovid tweets.

**Figure 7:**
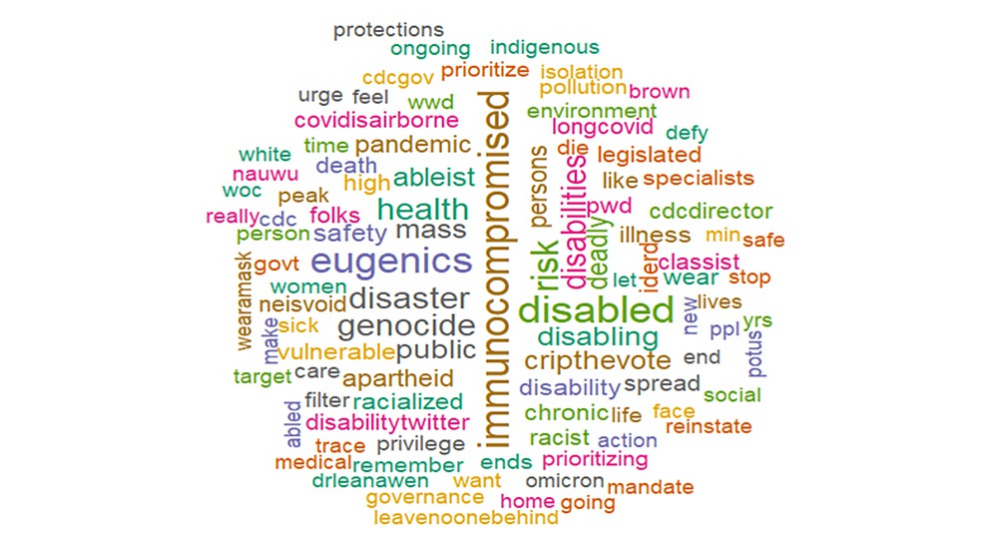
Word cloud of the top 100 negative #highriskcovid words.

**Figure 8:**
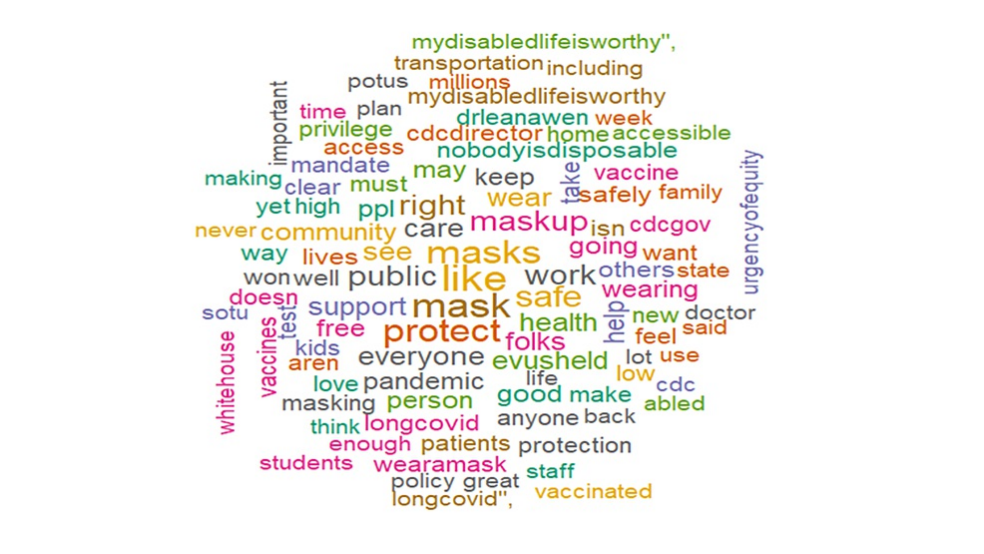
Word cloud of the top 100 positive #highriskcovid words.

We performed a sentiment analysis to better understand the nature of the collected tweets and deduce public perception (Figure 6). There was an overwhelming sense of negativity (19.27%) attributed to the opinions around high-risk COVID-19, contrasting sharply with the positive sentiments (14.88%) expressed. The analysis showed the expression of negative feelings (Figure 7) through keywords such as immunocompromised, eugenics, genocide, disaster, disabled, racialized, and death. While the analysis showed expression of positive feelings (Figure 8) through keywords such as masks, support, protect, vaccines, policy, and care.

**Figure 9:**
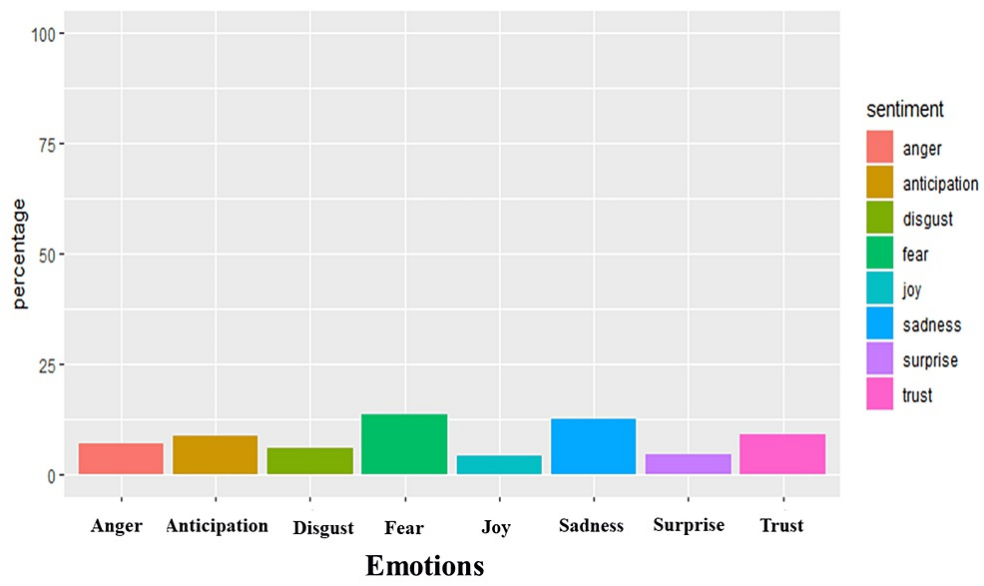
Emotional quotient analysis of #highriskcovid tweets. The Y axis is the percentage of sentiments of all analyzed tweets, while the X axis refers to the emotion in #highriskcovid tweets.

The emotional quotient of the sentiments was then examined using the NRC lexicon. The NRC lexicon comprises eight emotions, namely, anger, anticipation, disgust, fear, joy, sadness, and trust. Joy, surprise, trust, and anticipation are positive emotions, while anger, disgust, fear, and sadness are negative emotions. Figure 9 shows that tweets expressing emotions of fear (13.62%) were the most abundant, followed by tweets expressing sadness (12.47%) and trust (8.96%). The least emotions expressed were joy (4.3%), followed closely by surprise (4.42%) and disgust (5.65%). Overall, this result substantiates the deduction that people had an overwhelmingly pessimistic outlook toward high-risk COVID-19, with the predominant emotion being fear.

**Figure 10:**
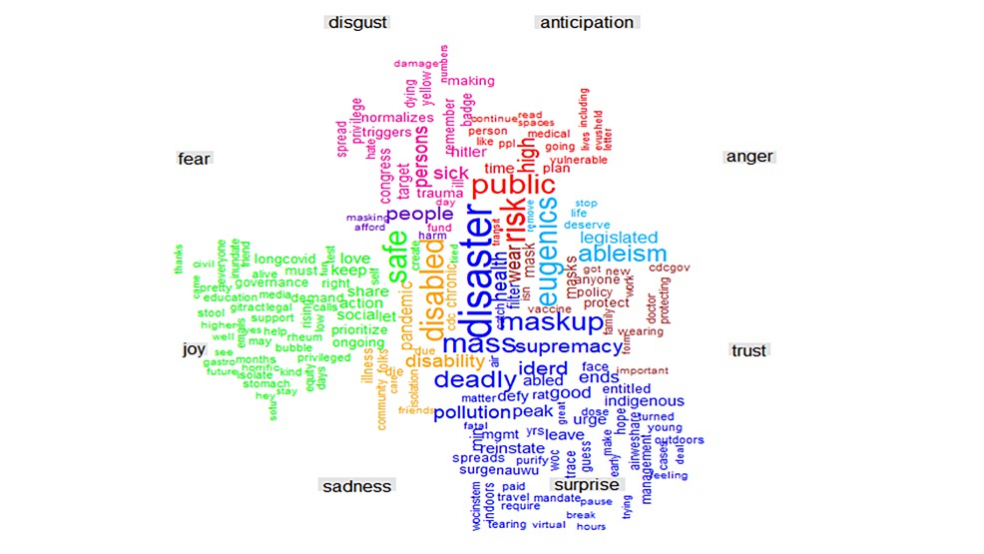
Comparative word cloud of the #highriskcovid words and the emotions they portray.

As illustrated in Figure 10, for the negative emotions, words such as spread, triggers, target, sick, and trauma were commonly used to denote fear, while keywords such as pandemic, isolation, disability, and chronic referenced sadness. Eugenics, ableism, and legislated were used about anger. For the positive emotions, the most frequently tweeted keywords such as doctor, protect, family, policy, vaccine, and treatment were related to the emotion of trust, while keywords such as governance, prioritize, action, and isolate were frequently tweeted in the context of joy. Similarly, risk, health, public, daily, plan, and medical reflected anticipation and disaster, dead, supremacy, virtual, and urge construed surprise.

Network analysis and topic modeling of high-risk COVID-19 tweets

**Figure 11:**
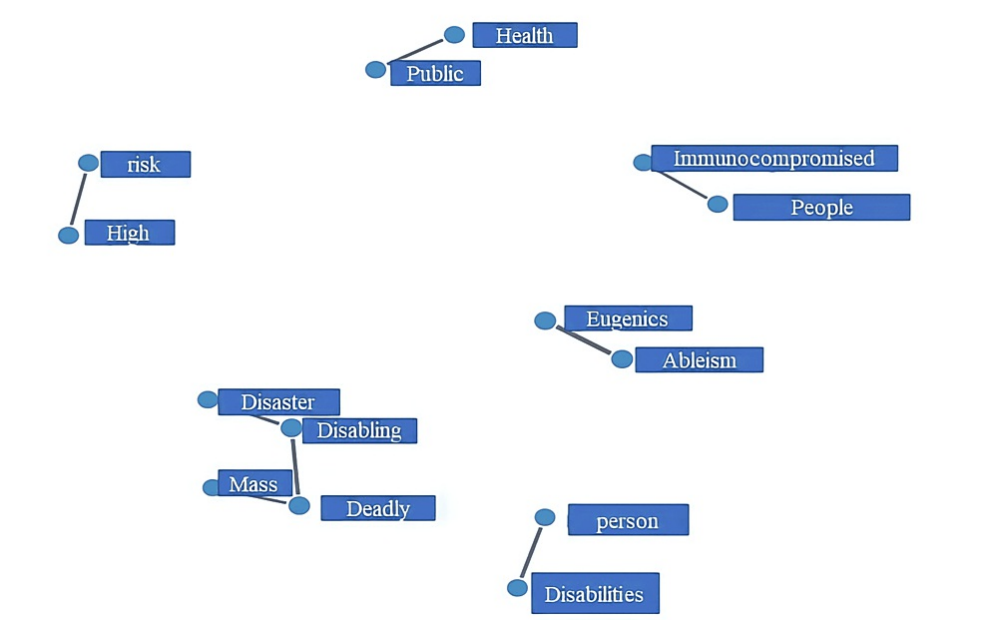
Network analysis of common co-occurring #highriskcovid words. The nodes represent words, while the edges (lines) are the weight of the connection between the nodes.

We identified words frequently co-tweeted and built a network model to visualize what words aggregated together in tweets (Figure 11). The largest network was a four-word association that included words like mass, disaster, deadly, and disabling. We also identified other co-tweeted words like high and risk, public and health, immunocompromised and people, mass and disaster, and deadly and disabling.

**Figure 12:**
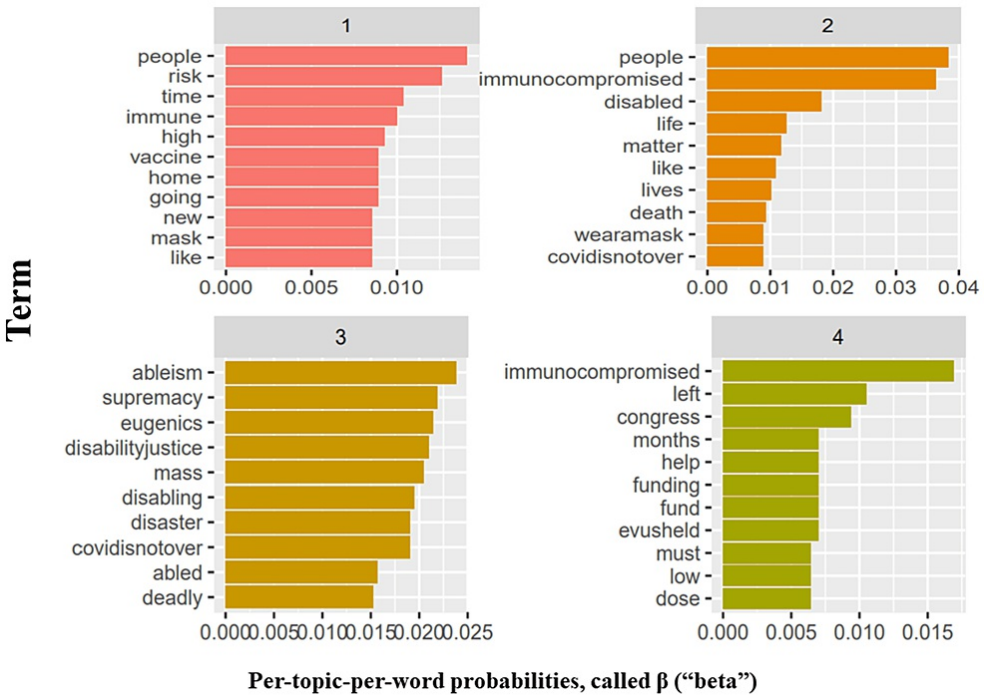
Modeled topics and their associated frequent terms among the analyzed #highriskcovid tweets.

**Figure 13:**
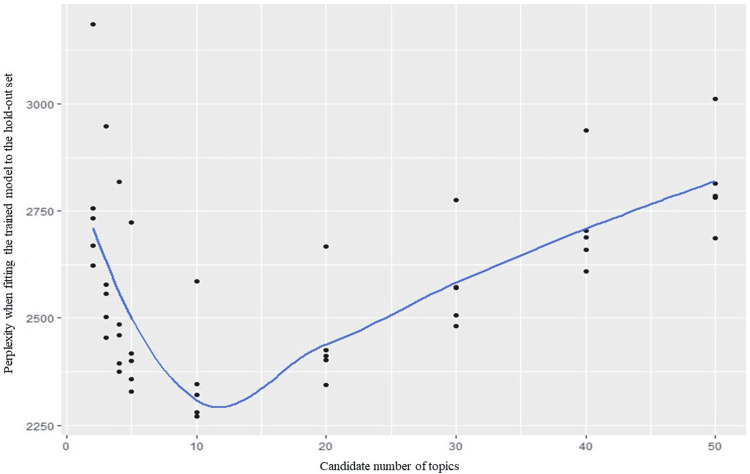
Five-fold cross-validation of topic modeling. The Y axis contains the perplexity score, while the X axis contains the candidate number of topics.  We identified a topic number between 10 to 17 to be the optimal range based on the perplexity score.

**Figure 14:**
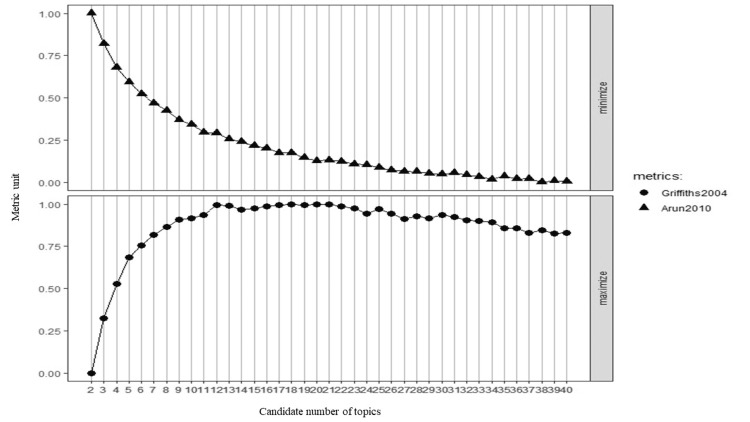
Result of LDAtuning using Griffiths2004 and Arun2010 metrics. The Y axis contains the metric unit, while the X axis contains the candidate number of topics. We identified a topic number between 10 to 26 (Griffith) and 10-25 (Arun) to be the optimal range based on the perplexity score.

Finally, we were curious to identify common high-risk COVID-19 topics in the analyzed tweets that would act as pointers to relevant themes most frequently discussed. To do this, we identified four topics based on topic coherence. The overall spectrum of potential discourse is represented according to the tweets analyzed in Figure 12. Topic 1 highlights people at high risk of COVID-19, topic 2 illustrates the protection of high-risk individuals, while topic 3 depicts the socio-political aspect of COVID-19.

## Discussion

Sentiment analysis is a natural language process analysis of open text and understanding of the attitudes portrayed by such texts (positive, negative, or neutral) using machine learning techniques [28]. Sentiment analysis has long found use in business, politics, finance, and other sectors but its use in healthcare is rising, particularly in epidemiology and disease outbreaks. However, it is still significantly less than in business and politics [29]. In this study, we focused on tweets in English on Twitter, most of which originated from English-speaking countries (the United States, Canada, and the United Kingdom). These three countries have some of the best economic indices and possess superior health infrastructure compared to the realities in other parts of the world [30,31]. Their representation in our analysis might be because these countries are more health-conscious than other English-speaking countries or are more likely to air their grievances on social media. It is possible that the inclusion of results from other languages, such as Chinese, Italian, and Spanish, in our analysis would have affected the result as all of these countries experienced high case fatality from COVID-19 infection [32]. However, translating from other languages to English results in translation loss and misrepresentation of information [33]. Most languages lack annotated sentiment analysis resources (a lexicon of sentiments and emotions, stopwords, etc.) which is critical for this analysis. In addition, some languages have a different coding style (non-Latin-based alphabets), making them difficult to pre-process. Most computer codes recognize Unicode [34].

Our analysis showed that two of the most frequently used words were immunocompromised and disabled which may denote the perception of the public as to who is classified as high risk for COVID-19. It was interesting to see that other previously described high-risk groups such as males, elderly, smokers, COPD patients, diabetic patients, and hypertensive patients were not frequently tweeted about. This might be because of inadequate knowledge of CEV (high-risk) individuals and points to a need to increase awareness about high-risk individuals. The most growing concern was about face masks and other preventive measures evidenced by the use of #maskup while #covidisnotover in reference to the ironic relaxation of the guidelines despite rising cases. Intriguingly, #eugenics was hugely used in tweets about high-risk COVID-19. Its use suggests the belief that COVID-19 is aimed to achieve “racial improvement” and produce “perfect human beings” eradicating the subsets of society (that high-risk group) deemed to have a greater predilection to severe COVID-19 infection. A bioethical dilemma in itself, its repetitive occurrence is borne out of the political discourse that the pandemic produced. Francis Galton (1822-1911), a British psychologist, was the first to use the term “eugenics” which translates to “well-born” and believed judicious mating gives the more suitable races or strains of blood a better chance of prevailing speedily over the less suitable [35]. It is apparent from our analysis that high-risk groups believe that present COVID-19 policies are jeopardizing their well-being to achieve herd immunity or sustainability of the general population.

Our sentiment analysis revealed that high-risk COVID-19 tweets were largely negative with words such as immunocompromised, eugenics, genocide, disaster, disabled, racialized, and death frequently being used. This highlights the belief that current preventive strategies may be discriminatory and do not advocate for those at the highest risk of more severe COVID-19 infections [36,37]. It spotlights the belief of some about the violation of the right to life and access to healthcare experienced due to existing government policies experienced by high-risk groups [38]. It lends to the belief by some that forcing high-risk groups to be exposed to the virus by relaxing lockdown measures is equivalent to genocide [39] and people should care enough to protect vulnerable people from dying by protecting themselves. Earlier in the pandemic, the United States and the United Kingdom ensured that high-risk groups were the first to receive vaccines and booster doses in addition to other support and care packages [40,41]. The prioritization of vaccination in high-risk groups is still obtainable and can be seen in the use of positive words like support, vaccines, masks, protect, policy, and care. These words affirm the belief in the efficacy of masking to reduce the risk of infection, the efficacy of vaccines, and the trust in the combinations of these measures to result in sound policy that catered to alleviate the concerns of high-risk COVID-19 groups. Support represents an appeal to improve the acceptance of individuals who had been reluctant about using masks or vaccinations. It could also be reflective of social campaigns to appreciate the health workers at the forefront of the pandemic with slogans such as “Protect the NHS” being common, inferring that following public health advice could reduce the disease burden and the service pressures the NHS was facing [42]. High-risk groups are known to have increased COVID-19-related fear and subjective risk perception regarding COVID-19 which affects their behavior [43] and, unsurprisingly, the prevalent emotions were fear, sadness, and trust. Fear revolved around spread, triggers, target, sick, and trauma.

Finally, we show that a few combinations of words are more frequently tweeted together: high and risk, public and health, immunocompromised and people, mass and disaster, deadly and disabling. All combinations highlight the importance of the concerns of CEVs. Our topic model identified 15 key clusters, of which we showed the most relevant four topics. The topics and their associated words vaguely represent sub-themes highlighting high-risk individuals (topic 1), sticking with preventive measures and protecting vulnerable populations (topic 2), talking about COVID-19 in the context of eugenics and supremacy (topic 3), and funding and prevention against COVID-19 (topic 4).

Our analysis has some strengths. We have used unsupervised machine learning to analyze curled tweets by adapting an analytic technique explored in the humanities to health. We also identified topic clusters, sentiments, and emotions that future studies should explore further. However, there were some limitations. We restricted our analysis to English-language tweets, as earlier explained. In addition, we focused on the one month following widespread governmental changes in COVID-19 policies. These limitations should be taken into account when interpreting our results.

## Conclusions

This research set out to gain insights into the state of mind and emotions felt by the minority who bear the greatest morbidity and mortality from COVID-19 at a time when governments are lifting bans and relaxing restrictions previously put in place to curb the spread of COVID-19. We observed that high-risk groups feel endangered and discriminated by current COVID-19 policies and have a growing concern about the rapidly disappearing COVID-19 preventive measures. Sentiments were markedly negative, with fear and sadness being the most predominant. While most people have returned to pre-COVID-19 life, those who bear the heaviest burden from COVID-19 still face fear and sadness. All relevant stakeholders (healthcare workers, caregivers, governmental, and non-governmental organizations) need to design and implement measures to educate the public about those at high risk, protect vulnerable individuals, and cater to their mental health needs as they face this unprecedented challenge.

## Appendices

**Table 1:** Clinically extremely vulnerable individuals (adapted from GOV.UK).

Clinically extremely vulnerable individuals
Solid organ transplant recipients
Active chemotherapy or immunotherapy for cancer
Hematological malignancies
Bone marrow or stem cell transplants in the last 6 months
Lung conditions especially severe lung diseases like all cystic fibrosis, severe asthma, and severe chronic obstructive pulmonary disease (COPD)
Congenital or acquired immunosuppressive conditions (such as severe combined immunodeficiency (SCID), homozygous sickle cell, down’s syndrome
Renal disease especially on dialysis or with chronic kidney disease (stage 5)
Pregnant women especially those with significant heart disease
Neurological conditions (such as Parkinson’s disease, motor neuron disease, multiple sclerosis, or cerebral palsy)
Diabetes
Heart disease
Other people who have also been classed as clinically extremely vulnerable based on clinical judgment
Hypertension
